# The Therapeutic Efficacy of Curcumin vs. Metformin in Modulating the Gut Microbiota in NAFLD Rats: A Comparative Study

**DOI:** 10.3389/fmicb.2020.555293

**Published:** 2021-01-14

**Authors:** Ruifang Li, Yurong Yao, Pengfei Gao, Shurui Bu

**Affiliations:** ^1^Department of Gastroenterology, Jinshan Hospital Affiliated to Fudan University, Shanghai, China; ^2^Department of Infection, Jinshan Hospital Affiliated to Fudan University, Shanghai, China; ^3^Department of Traditional Chinese Medicine, Jinshan Hospital Affiliated to Fudan University, Shanghai, China

**Keywords:** non-alcoholic fatty liver disease, curcumin, metformin, gut microbiota, short-chain fatty acids

## Abstract

Structural disruption of gut microbiota is closely related to the occurrence of non-alcoholic fatty liver disease (NAFLD). Previous research has demonstrated that both curcumin (CUR) and metformin (MET) have a therapeutic effect against NAFLD and play a role in modulating the gut microbiota. However, there is a lack of direct comparison between the two medications in terms of the therapeutic efficacy and the regulatory effect on gut microbiota. In this study, we administered either CUR or MET to rats with high-fat diet (HFD)-induced obesity to observe changes in body parameters, biochemical parameters, liver, and ileum pathology and gut microbiota, and used next generation sequencing and multivariate analysis to evaluate the structural changes of gut microbiota in a NAFLD rat model before and after CUR and MET intervention. It was found that both CUR and MET attenuated hepatic ectopic fat deposition, alleviated inflammatory factors, and improved intestinal barrier integrity in HFD-fed rats. More importantly, CUR and MET reduced the *Firmicutes/Bacteroidetes* ratio and reverted the composition of the HFD-disrupted gut microbiota. Both CUR and MET treatments effectively modified the gut microbiome, enriched the abundance of beneficial bacteria and reduced opportunistic pathogens in obese rats. The abundance of *Butyricicoccus* was increased while the abundance of *Dorea* was decreased in HFD + CUR group. Besides, some beneficial bacteria such as *Prevotella* were increased in MET-treated animals. Spearman’s correlation analysis showed that *Helicobacter, Akkermansia, Desulfovibrio, Romboutsia, Corynebacterium, Lactobacillus, Ruminococcaceae_*unclassified, *Lachnospiraceae_unclassified*, and *Clostridiales_unclassified* showed significantly positive correlations with TG, TC, LDL-C, GLU, IL-6, IL-1β, and TNF-α, and negative correlations with HDL-C (both *p* < 0.05). However, *Prevotella* and *Stomatobaculum* showed an opposite trend. In summary, CUR and MET showed similar effects in alleviating hepatic steatosis, improving intestinal barrier integrity and modulating gut microbiota in HFD-induced obesity rats, and therefore may prove to be a novel adjunctive therapy for NAFLD.

## Introduction

Non-alcoholic fatty liver disease (NAFLD) is one of the most common chronic liver diseases, with a prevalence varying from 25 to 40% worldwide (Younossi et al., 2016). It is characterized by increased triglyceride (TG) accumulation in hepatocytes (Younossi et al., 2018). NAFLD is a clinical pathological syndrome ranging from non-alcoholic simple fatty liver (NAFL) to non-alcoholic steatohepatitis (NASH), which can progress to liver cirrhosis and hepatocellular carcinoma (HCC) (Abdul-Hai et al., 2015). The occurrence of NAFLD is associated with obesity, dyslipidemia, insulin resistance (IR), type 2 diabetes mellitus (DM2), and cardiovascular disease (Byrne et al., 2009). No approved drugs are currently available for the prevention and treatment of NAFLD, highlighting the extreme importance to develop potentially effective therapeutic targets for it (Sumida and Yoneda, 2018).

The human gut microbiota, composed of over trillions of bacteria with hundreds of species, has recently been recognized to be closely related to the development of NAFLD owing to the advancement of the “intestinal-hepatic axis” theory and the advent of gene sequencing technology (Leung et al., 2016). Animal studies have demonstrated that germfree mice fed on high-fat and high-sugar diets did not become obese, but the insulin and body fat levels were increased after colonizing the cecal microbiota in obese mice (Abdou et al., 2016). In addition, new evidence has shown that change in the gut microbiota composition can influence nutrient acquisition and energy regulation of the host, which could result in IR and diabetes (Ma et al., 2017). This indicates that the gut microbiota plays a key role in the development and progression of NAFLD, and modulating the gut microbiota may be a novel strategy for NAFLD prevention. Accordingly, targeting the gut microbiota by using prebiotics, probiotics, or other dietary interventions and drugs could be an effective way to prevent or alleviate NAFLD (Meroni et al., 2019). Curcumin (CUR) is the main active component of turmeric and has proved to be effective in preventing obesity, diabetes, and inflammation (Carrera-Quintanar et al., 2018). CUR supplementation is reportedly effective in alleviating hepatic steatosis via modulating lipid metabolism such as cholesterol, TG, and free fatty acid (FFA) concentrations (Ferguson et al., 2018), and improving IR in high-fat-diet (HFD)-fed obese rats (Niu et al., 2019). Another study provided new evidence that CUR supplementation significantly shifted the ratio between beneficial and pathogenic microbiota (Feng et al., 2017). These findings suggest that CUR has the potential to cure dyslipidemia and NAFLD. However, the efficacy of CUR on fatty liver disease remains largely unknown.

Unlike CUR, metformin (MET) has a relatively higher oral bioavailability and its mechanism in treating metabolic diseases by activating the AMP-activated protein kinase (AMPK) pathway has been well established (Rena et al., 2017). However, recent studies have shown that intestinal microbiota may be an important target of MET. In their study of HFD-induced obesity rats, Zhang et al. (2015) found that MET could increase the number of short-chain fatty acid-producing bacteria, reduce intestinal biodiversity, and pose a positive effect on beneficial bacteria such as *Prevotella* and *Lactobacillus.* Metagenomic and metabolomic analyses of newly diagnosed DM2 samples *in vivo* and *in vitro* experiments by Sun et al. (2018) showed that MET played a role in improving metabolism by reducing *Baeteroides fragilis* in microbiota, increasing the level of glycoursodeoxycholic acid and tauroursodeoxycholic acid and inhibiting intestinal farnesoid X receptor (FXR), which may provide a new drug target for the treatment of obesity-related metabolic diseases.

Previous studies have proved that both CUR and MET play a role in treating NAFLD and modulating the gut microbiota. However, there is still a lack of direct comparison of the effects of these two drugs. In this study, we used next generation sequencing (NGS) technique and multivariate analysis to compare the structural changes of gut microbiota after treatment with CUR and MET in NAFLD rats, with the aim to provide a direct comparison between CUR and MET in modulating the gut microbiota structure during the treatment of NAFLD rats, thus confirming the possibility of using CUR as a novel adjunctive therapy for NAFLD.

## Materials and Methods

### Drugs and Diets

CUR, MET, and 0.5% sodium carboxymethylcellulose (NaCMC) used in this study were all purchased from Sigma Chemical Co., United States. HFD diet (composed of 88% normal chow diet, 10% lard oil, 1.5% cholesterol, and 0.5% bile salt) and normal chow diet (composed of 15.4% fat, 25.8% protein, and 58.8% carbohydrate, totaling 3.856 kcal/g calories) were obtained from BOAIGANG Biological Technology Co., LTD., Beijing, China.

### Animal Experiments

Thirty-two specific pathogen-free (SPF) male Sprague Dawley rats aged 6 weeks and weighing 170–190 g (Jiesijie Experimental Animal Co., LTD, Shanghai, China) were housed 4 per cage under a strictly controlled temperature of 22 ± 2°C with 12 h light/dark cycle with free access to food and water. After 2 weeks acclimatization, the rats were randomized to a normal control (NC) group (*n* = 8) and a HFD group (*n* = 24). Animals in NC group were regularly fed with the conventional rat normal chow diet, and those in HFD group were fed with HFD diet. After 10 weeks feeding, one rat was randomly selected from NC group and 3 from HFD group to investigate the adipohepatic degrees of the liver. The remaining 21 rats in HFD group were further equally randomized into a HFD group, a HFD + CUR (200 mg/kg/day) group, and a HFD + MET (200 mg/kg/day) group. CUR was prepared in a solution of 0.5% NaCMC. Therefore, all rats in NC and HFD subgroups were given the corresponding volume of 0.5% NaCMC.

The experiment was continued for 14 weeks and the body weight of each rat was recorded weekly. Stool samples were collected at 0, 10, and 14 weeks, frozen in liquid nitrogen, and stored at −80°C for further analysis.

At the end of the experiment, all the rats were anesthetized by 10% chloral hydrate and sacrificed after fasting for 12 h. The body weight was measured and blood was freshly collected by cardiac puncture and serum was stored at −80°C after centrifugation at 3,000 r/min for 10 min. The liver tissue was weighed, and the intestine and feces samples were frozen in liquid nitrogen and kept at −80°C for further analysis. Part of the liver and intestine tissues were either fixed in 4% formalin or 2.5% glutaraldehyde for histopathological analysis.

All animal studies were conducted in strict accordance with the ethical guidelines and approved by the Research Animal Care Committee of Jinshan Hospital affiliated to Fudan University, China.

### Serum Biochemical Analysis

The serum levels of alanine amino transferase (ALT), aspartate aminotransferase (AST), total cholesterol (TC), TG, high-density lipoprotein cholesterol (HDL-C), low-density lipoprotein cholesterol (LDL-C), and glucose (GLU) were biochemically analyzed (Hitachi, Japan). In addition, the concentrations of inflammatory factors including tumor necrosis factor-α (TNF-α), Interleukin-1β (IL-1β), IL-6, IL-17, IL-23, interferon-inducible protein-10 (IP-10), and OX-40 were detected using enzyme-linked immunosorbent assay kits according to the instructions of the manufacturer (Mlbio, Shanghai, China).

### Histopathological Assessment of the Liver and Intestinal Tissues

The liver and ileal tissues of the same part from each rat were fixed in 4% paraformaldehyde, dehydrated in ethyl alcohol, dealcoholized in xylene, paraffin embedded, sliced into 3–4 μm sections, stained with hematoxylin and eosin (H&E), and finally observed under the light microscope. Then the stained liver was analyzed histologically by the novel NAFLD activity score (NAS) system to grade the steatosis, ballooning and inflammation of the liver tissue, with a consultation to a guide on the diagnosis and treatment for NAFLD (Bechmann et al., 2013). To determine hepatic lipid accumulation, liver sections were also stained with Oil Red O and counterstained with hematoxylin. Furthermore, the Olympus Image-Pro Plus 6.0 software was utilized to perform quantitative analysis and calculate the stained Oil Red O areas.

For transmission electron microscopy (TEM), ileal tissues were cut into 1 mm^3^ sections and fixed with 2.5% buffered glutaraldehyde, and post-fixed with 1% osmium tetroxide for 1 h at 4°C, dehydrated through a gradient series of alcohol, then embedded in epoxy resin. Samples were sliced into 80 nm sections and stained with uranyl acetate and lead citrate. Images were examined using a transmission electron microscope (JEOL Ltd, Tokyo, Japan).

### DNA Extraction and 16S rDNA Next Generation Sequencing

Fecal samples were stored at -80°C until DNA extraction. The DNA was extracted from 250 mg samples using the E.Z.N.A. DNA Stool Kit (Omega Bio-tek, Inc., GA, United States) following the manufacturer’s instructions. The V3–V4 region of 16S rRNA was selected for high-throughput sequencing (95°C for 3 min, 94°C for 30 s, 58°C for 30 s, 72°C for 30 s, 72°C for 5 min). The forward primer (341F) was 5′-CCTACGGGNGGCWGCAG-3′ and the reverse primer (805R) was 5′-GACTACHVGGGTATCTAATCC-3′. The first PCR reaction was carried out in a compound containing 5× buffer 10 μL, 1 μL dNTP (10 mM), 1 U Fast Pfu DNA polymerase, 1 μL of each primer (10 mM), and 20–50 ng template DNA by addition of ultra pure water to 50 μL. The reaction DNA concentration and purity were checked by running the samples on 1% agarose gels, and 2% agarose gel electrophoresis was used to extract samples with good detection results. All PCR products were purified by Axy PrepDNA Gel Extraction Kit (Axygen Biosciences, Union City, CA, United States), and FTC-3000 Real-Time PCR (Funglyn Biotech, Ontario, Canada) was used for fluorescence quantification. After homogenization, the libraries were constructed, and sequencing was completed via the MiSeq 600 cycle platform (Illumina Inc., CA, United States) in Mobio Biomedical Technology Co., LTD (Shanghai, China).

### Bioinformation Analysis

The effective sequences of each sample were obtained by bar code and the low-quality sequences at the end of the sequencing result were removed using QIIME software. According to the overlapping relationship between paired-end readings, the paired reads were spliced into a sequence by FLASH software (Magoč and Salzberg, 2011) obtained from http://www.cbcb.umd.edu/software/flash, and then Mothur software (version 1.33.3) (Schloss et al., 2009) obtained from http://www.mothur.org was used to control the quality of the sequences, so as to get the optimized sequence, and then clustered into OTUs (operational taxonomic units) at a 97% similarity level. Based on the classification information, the community structure was analyzed at the six classification levels of phylum, class, order, family, genus, and species.

Based on the above analysis, a series of statistical and visual mapping of bacterial community and system development (UniFrac) were carried out. The alpha-diversity analysis (such as Chao, ACE, Shannon, and Simpson), Beta-diversity analysis (unweighted UniFrac analysis) as well as Rarefaction analysis were carried out by using the Mothur software and plotted by R project (version 3.2.3). Based on OTU abundance and distance, R project was used for visualization of bacterial community classification and distribution such as principal coordinate analysis (PCoA), principal component (PCA), and heatmap mapping. To detect the heterogeneity characteristics among the groups, linear discriminant analysis effect size (LEfSe) was used as well as non-parametric factorial Kruskal-Wallis (KW) sum-rank test.

### Statistical Analysis

All the data (except for microbial community analysis) were analyzed with SPSS 23.0 (IBM Corp., Armonk, NY, United States) and expressed as the mean ± *SD*. The mean value of each group was analyzed by One-way ANOVA analysis. The differences between two groups were examined by Student’s *t*-test. Experiments were repeated ≥ 3 times and *P* < 0.05 was considered to be statistically significant.

## Results

### Attenuating Effects of CUR and MET on Hepatic Fat Deposition

**FIGURE 1:**
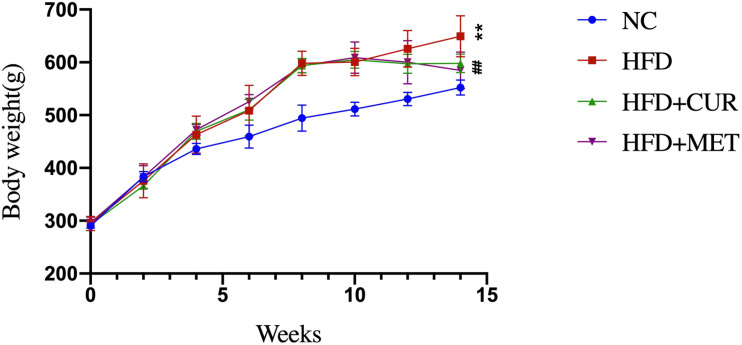
Effects of CUR and MET on body weight. Seven rats per group (*n* = 7). Values were presented as the means ± *SD* for each group. *p < 0.05, **p < 0.01 compared with the NC group. ^#^*p* < 0.05, ^##^*p* < 0.01 compared with the HFD group.

**FIGURE 2:**
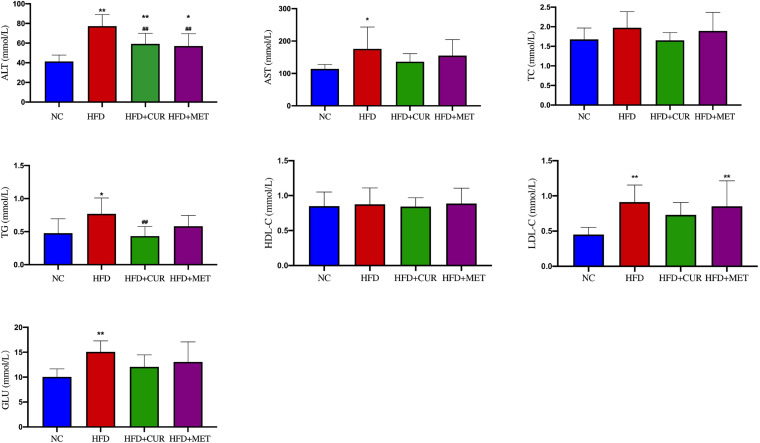
Influence of CUR and MET on the serum biochemical index. Seven rats per group (*n* = 7). Values were presented as the means ± *SD* for each group. *p < 0.05, **p < 0.01 compared with the NC group. ^##^*p* < 0.01 compared with the HFD group.

**FIGURE 3:**
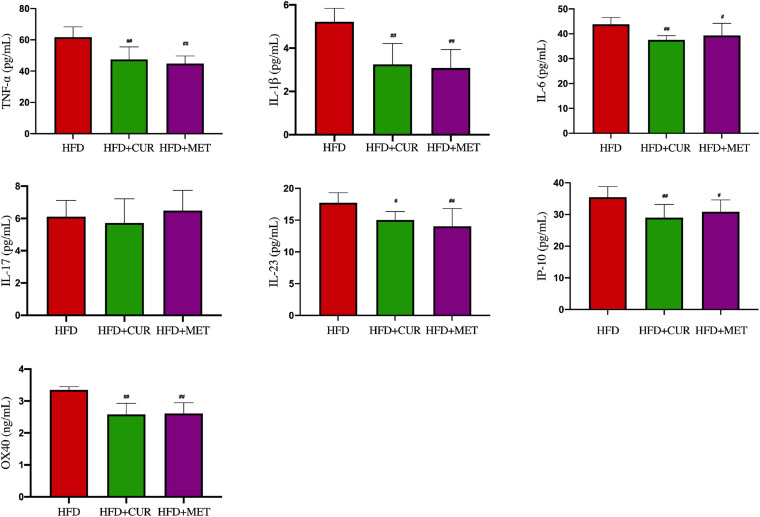
Influence of CUR and MET on the serum inflammation factors. Seven rats per group (*n* = 7). Values were presented as the means ± *SD* for each group. ^#^*p* < 0.05, ^##^*p* < 0.01 compared with the HFD group.

After 10 weeks feeding with HFD, four rats (one from NC group and 3 from HFD group) were randomly selected to investigate pathological changes of liver. The results of H&E staining as well as the NAFLD activity score showed that NAFLD was induced in the rats successfully (Supplementary Figure S1 and Supplementary Table S1). The mean body weight of rats in HFD group was significantly higher than that in NC group (*p* < 0.01, Figure 1). As shown in Figure 2, serum levels of ALT, AST, TG, LDL-C, and GLU in HFD group were significantly higher than those in NC, HFD + CUR, and HFD + MET groups. However, the concentration of HDL-C showed an opposite tendency as compared with ALT, AST, TG, and LDL-C. Compared with HFD group, serum levels of TNF-α, IL-1β, IL-6, IL-23, IP-10, and OX40 were reduced significantly after CUR or MET treatment (*p* < 0.05, Figure 3), but these levels in HFD + CUR group were similar to those in HFD + MET group (*p* > 0.05).

**FIGURE 4:**
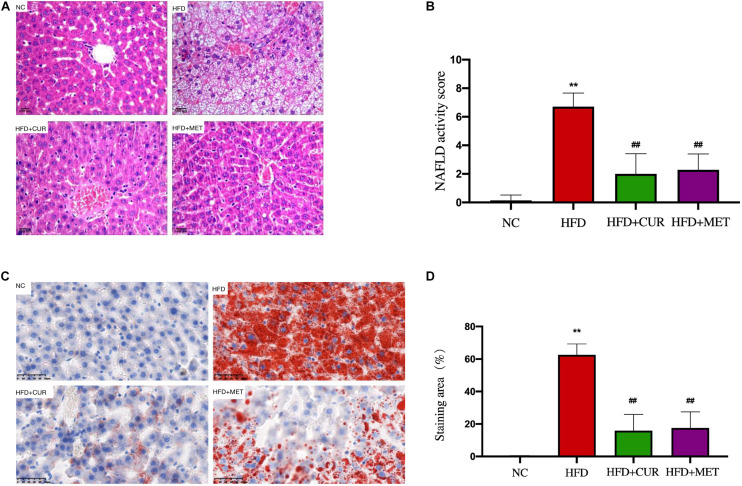
Effects of CUR and MET on HFD-induced hepatic steatosis. **(A)** HE-stained liver tissue. **(B)** Score of liver HE staining. **(C)** Oil red O-stained liver tissue. **(D)** The quantitative results of the oil red O staining from rats in the NC, HFD, HFD + CUR, and HFD + MET groups. Seven rats per group (*n* = 7). Values were presented as the means ± *SD* for each group. **p < 0.01 compared with the NC group. ^##^*p* < 0.01 compared with the HFD group.

H&E staining demonstrated a disordered structure of the hepatic lobules all over the liver with obvious lipid droplet accumulation and inflammatory cell infiltration in HFD group (Figure 4A), while the number of lipid vacuoles in HFD + CUR group and HFD + MET group was decreased significantly as compared with HFD group (Figures 4A,B). Oil-Red O staining demonstrated a decrease in lipid accumulation in HFD + CUR and HFD + MET groups compared with HFD group (Figures 4C,D). All these results clearly demonstrated that CUR and MET could attenuate hepatic fat deposition in NAFLD rats induced by HFD and inhibit liver inflammation.

### Ameliorating Effects of CUR and MET on Intestinal Barrier Integrity

**FIGURE 5:**
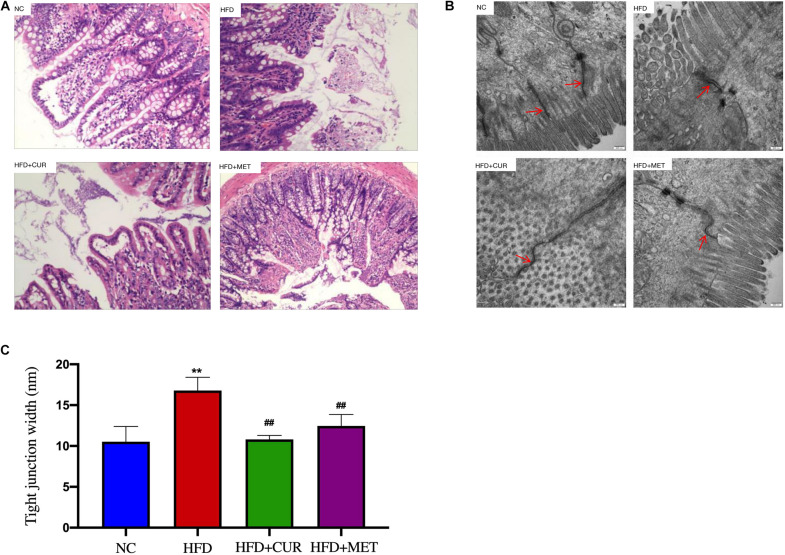
Influence of CUR and MET administration on intestine. **(A)** HE-stained Ileal tissue. **(B)** Ultrastructural observation of the tight junctions in the ileal mucosa (transmission electron microscopy, 50,000×). **(C)** Width of the tight junction gap. Seven rats per group (*n* = 7). Values are presented as the means ± *SD* for each group. **p < 0.01 compared with the NC group. ^##^*p* < 0.01 compared with the HFD group.

H&E staining of the intestinal tissues showed that the intestinal villus was short, sparse and slightly damaged and inflammatory cell infiltration was seen in the intestinal mucosa in HFD group. In contrast, these morphological changes were ameliorated after CUR and MET treatment (Figure 5A). To evaluate the damage to the ileal microstructure, tight junctions of the rat ileum were observed by transmission electron microscopy. The intact tight junctions of the rat ileum in NC group were widened after HFD feeding, but restored to the normal state after CUR or MET treatment (Figures 5B,C). These results suggest that CUR and MET could ameliorate intestinal barrier integrity in NAFLD rats.

### Analysis of High-Throughput Sequencing Results

To study the changes of gut microbiota in rats, a total of 54 fecal samples were collected from the HFD, HFD + CUR, and HFD + MET groups at 0, 10, and 14 weeks and they were marked as HFD (0W), HFD (10W), HFD (14W), HFD + CUR (14W), and HFD + MET (14W) groups. It means that fecal samples of only six rats out of seven in each feeding category were subjected to the time-course high-throughput sequencing analysis. High throughput sequencing of 16S rDNA by the next generation sequencer produced 2,697,855 high-quality sequences from 54 fecal samples collected at 0, 10, and 14 weeks, with a mean of 49,960 sequences per sample. High-quality sequences were then delineated into 1,510 OTUs at a similarity cutoff of 97%. Shannon and Rarefaction analysis showed that most of the gut microbial organisms in each sample could be captured at the current sequencing depths (Supplementary Table S2 and Supplementary Figures S2, S3). Compared with the HFD (0W) group, the Shannon diversity index and Chao index in HFD (10W) group were significantly lower (*p* < 0.01), and there was no significant difference in Shannon diversity index and Chao index between the HFD (0W) and HFD (14W) groups. In addition, the data showed no significant difference in richness (as indicated by rarefaction OTU estimates) and diversity between HFD (14W), HFD + CUR (14W) and HFD + MET (14W) groups (Table 1).

**TABLE 1:** The abundance of OTUs, Chao1 index and Shannon index in each group.

Group	HFD (0W) *N* = 18	HFD (10W) *N* = 18	HFD (14W) *N* = 6	HFD + CUR (14W) *N* = 6	HFD + MET (14W) *N* = 6
OTUs	657 ± 17	537 ± 23**	624 ± 28	621 ± 9	571 ± 16**
Chao index	771 ± 17	650 ± 22**	706 ± 32	731 ± 13	685 ± 19**
Shannon index	4.33 ± 0.07	3.83 ± 0.09**^#^	4.36 ± 0.14	4.71 ± 0.14*	4.20 ± 0.15

*p < 0.05, **p < 0.01, vs. HFD (0W); ^#^p < 0.05, vs. HFD (14W).

### Response of the Gut Microbiota Structure to HFD

**FIGURE 6:**
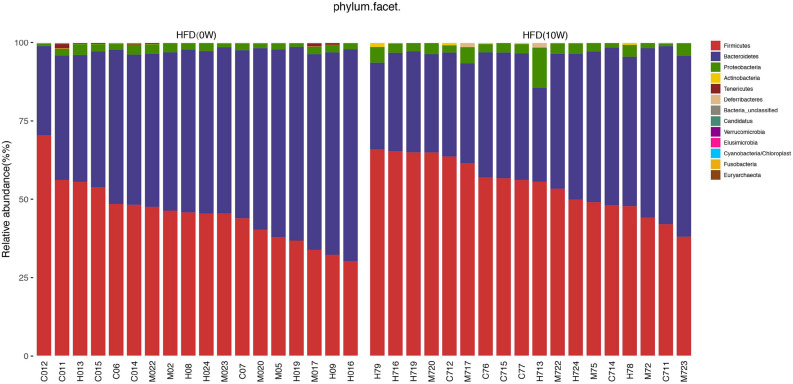
Responses of the structure of the gut microbiota to HFD at the phylum level.

**FIGURE 7:**
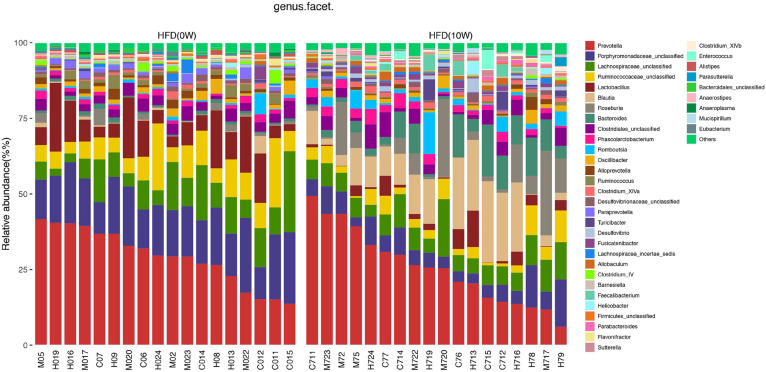
Responses of the structure of the gut microbiota to HFD at the genus level.

The composition of the gut microbiota at both phylum and genus levels was analyzed to determine which types of bacteria were affected by HFD. At the phylum level, the main components of the microbiota were *Bacteroides, Firmicutes, Proteobacteria*, and *Verrucomicrobia*, accounting for more than 90% of the total. Compared with HFD (0W) group, an increase in *Firmicutes, Proteobacteria*, and *Actinobacteria* and a decrease in *Bacteroidetes* and *Tenericutes* were observed in HFD (10W) group, as well as a significantly higher *Firmicutes-*to*-Bacteroidetes* (F/B) ratio (*p* < 0.05) (Figure 6). At the genus level, the composition of the gut microbiota changed greatly between the two groups. Compared with HFD (0W) group, the abundance of *Porphyromonadaceae_unclassified, Ruminococcaceae_unclassified, Lactobacillus, Ruminococcus, Bacteroidale_unclassified, Alistipes*, and *Anaeroplasma* was reduced, and the abundance of *Blautia, Roseburia, Allobaculum, Desulfovibrio, Anaerostipes*, and *Romboutsia* was increased in HFD (10W) group (Figure 7).

### Altering the Gut Microbiota Structure by CUR and MET in HFD-Fed Rats

**FIGURE 8:**
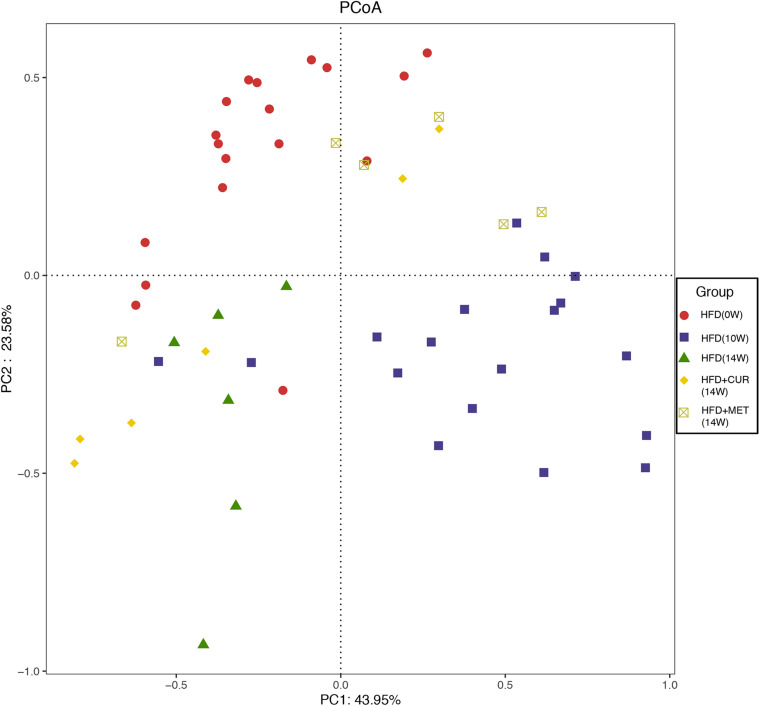
PCoA score plot based on unweighted Unifrac metrics.

Unifrac distance-based principal coordinate analysis (PCoA) was utilized to analyze the overall structural change of the gut microbiota. Both weighted and unweighted Unifrac analysis showed overt changes of the overall gut microbial community in response to CUR and MET intervention. Compared with weighted assessment (Supplementary Figure S4), unweighted Unifrac analysis exhibited a greater distinction (Figure 8). PCoA of β-diversity in the gut microbiota showed that the gut microbiota of HFD (10W) and HFD (14W) groups was completely separated from that of the HFD (0W) rats during the HFD intervention. However, the gut microbiota of the rats in HFD + CUR and HFD + MET groups was completely separated from that of HFD group at the end of the experiment (14W), and the distance of the gut microbiota in HFD (0W), HFD + CUR and HFD + MET groups was shorter than that in HFD (10W) and HFD (14W) groups. In summary, the structure of the gut microbiota was altered after HFD feeding, and partly reversed after CUR or MET treatment.

### Modulating Effects of CUR and MET on the Key Phylotype of Gut Microbiota

**FIGURE 9:**
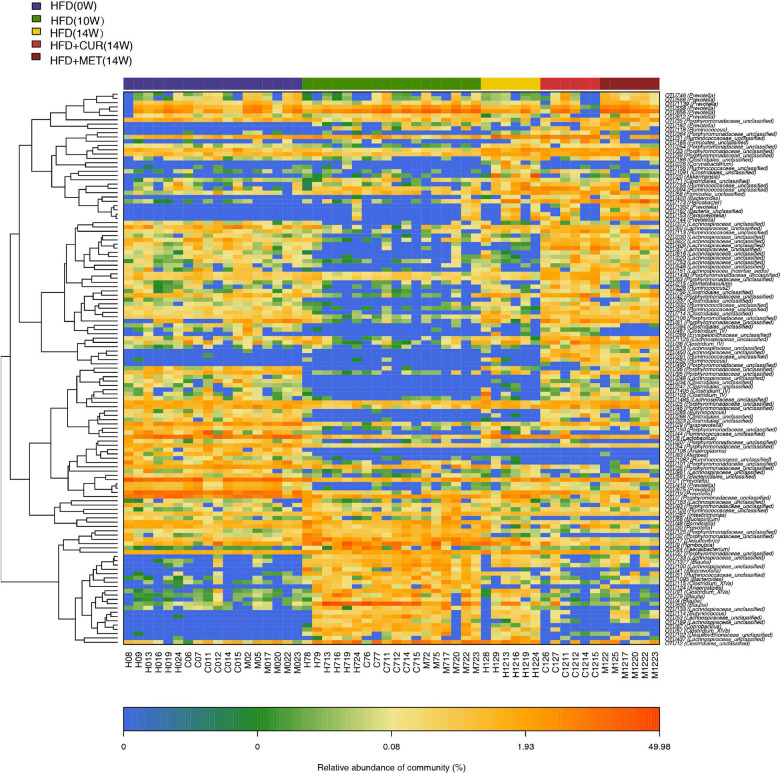
Responses of the structure of the gut microbiota to HFD, CUR and MET treatment. Heatmap of the abundance of the OTUs in the HFD (0W), HFD (10W), HFD (14W), HFD + CUR (14W), and HFD + MET (14W) groups. Red and blue colors indicate the abundance of OTUs that were more and less abundant.

**FIGURE 10:**
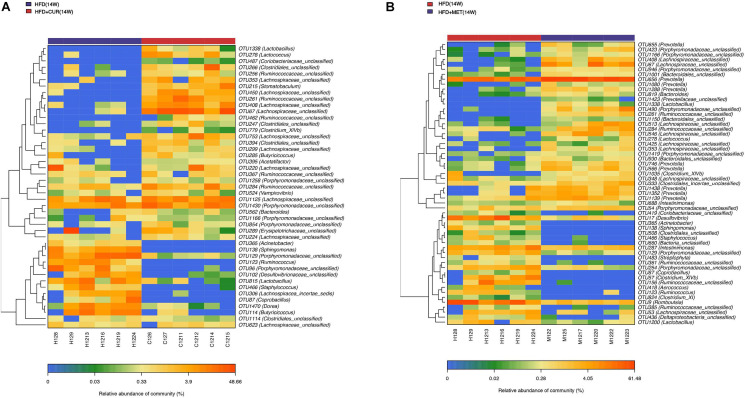
Responses of the structure of the gut microbiota to CUR and MET treatment. **(A)** The changing direction of the OTUs by CUR. **(B)** The changing direction of the OTUs by MET. Red and blue colors indicate the abundance of OTUs that were more and less abundant, respectively, in HFD + CUR (14W) group and HFD + MET (14W) group relative to HFD (14W) group.

The community heatmap diagram showed that compared with HFD (14W) group, 31 OTUs were enriched and 14 OTUs were decreased by CUR, while 34 OTUs were increased and 23 OTUs were decreased by MET. Both CUR and MET enriched the abundance of *Lactobacillus* (OTU1338), *Lactococcus* (OTU278), *Bacteroides* (OTU562 and OTU 819), *Clostridium_XIVb* (OTU1035 and OTU 779), *Ruminoccoccaceae_ unclassified* (OTU261, OTU 462, OTU 285, OTU 367, and OTU 284), *Porphyromonadaceae_unclassified* (OTU1430, OTU 1258, OTU 1166, OTU 854, OTU 423, OTU946, OTU490, OTU1419, and OTU54) and *Lachnospiraceae_unclassified* (OTU353, OTU 408, OTU 67, OTU 753, OTU 299, OTU 220, OTU 1125, OTU 224, OTU 513, OTU 546, OTU 425, and OTU 348), whereas they decreased the abundance of *Acinetobacter* (OTU365), *Sphingomonas* (OTU138), *Porphyromonadaceae_unclassified* (OTU129), *Coprobacillus* (OTU87), *Ruminococcus* (OTU123), *Desulfovibrio* (OTU17 and OTU102), and *Staphylococcus* (OTU466) (Figure 9 and Supplementary Figure S5). Notably, the abundance of *Butyricicoccus* (OTU285), *Stomatobaculum* (OTU215), *Acetatifactor* (OTU395), and *Vampirovibrio* (OTU524) was increased, while the abundance of *Dorea* (OTU1470) was decreased in response to CUR treatment (Figure 10A). The abundance of *Prevotella* (OTU655, OTU 656, OTU 1080, OTU 1288, OTU 746, OTU 566, OTU 1438, OTU1352, and OTU1139) was markedly increased, while the abundance of *Streptophyta* (OTU483), *Aerococcus* (OTU418), and *Romboustia* (OTU9) was decreased in response to MET treatment (Figure 10B).

**FIGURE 11:**
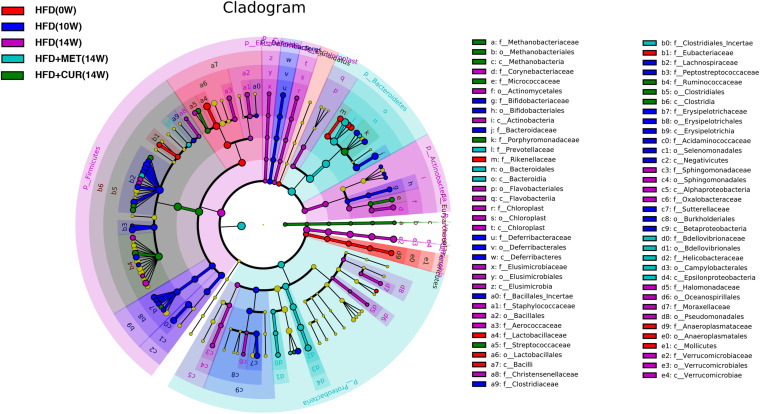
Key bacterial alterations in response to the HFD, CUR, and MET treatments. Cladogram generated by the LEfSe analysis shows enriched taxa in feces from the HFD (0W, red), HFD (10W, blue), HFD (14W, purple), HFD + CUR (14W) (green), and HFD + MET (14W) (cyan) groups.

Linear discriminant-based analysis (LDA) was utilized to further identify significant changes in gut microbial compositions in response to both CUR and MET administration. The taxonomic cladogram generated by LEfSe exhibited different species in HFD (0W), HFD (10W), HFD (14W), HFD + CUR (14W), and HFD + MET (14W) groups. The comparison between HFD (14W) and HFD + CUR (14W) groups showed that the abundance of *Desulfobaculum, Ruminococcus2, Methanosphaera*, and *Erysipelotrichaceae_incertae_sedis* was significantly increased after CUR intervention. Compared with HFD (14W) group, MET administration significantly increased the abundance of *Bacteroidales, Prevotella, Paraprevotella, Bdellovibrionales, Prevotellaceae, Prevotella*, and *Bdellovibrionaceae*, all of which belong to *Bacteroidetes*. Besides, the abundance of *Lachnospiracea_incertae_sedis, Clostridiales_Incertae*, and *Lactococcus* belonging to *Firmicutes*, and *Proteobacteria* including *Vampirovibrio* and *Pasteurella* was also increased. Interestingly, LEfSe exhibited that both CUR and MET had an inhibitory effect on *Lactobacillales, Aerococcus, Staphylococcus*, and *Streptococcus*, which belong to the class of *Bacilli*, as well as *Verrucomicrobiaceae, Romboutsia, Akkermansia* compared with HFD (14W) group (Figure 11). These results suggest that both CUR and MET could reverse the composition of gut microbiota in obese rats induced by HFD and make it similar to that of the normal rats.

### Associations Between the Gut Microbiota Altered by CUR/MET and Host Metabolic Parameters

**FIGURE 12:**
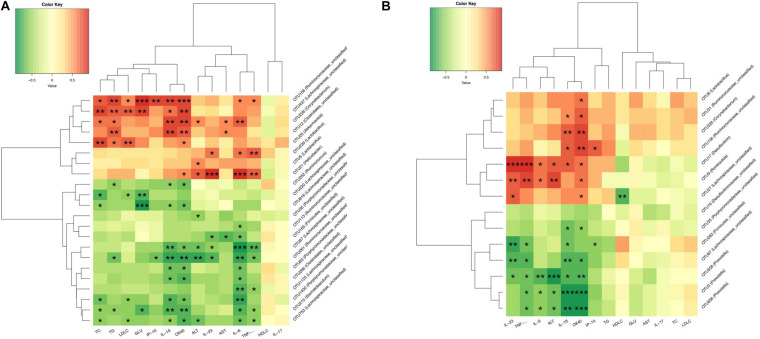
CUR and MET-altered OTUs that were significantly correlated with host metabolic parameters. Spearman’s correlation analysis was performed between the OTUs which had significant difference among the HFD (14W), HFD + CUR (14W), and HFD + MET (14W) groups and specific metabolic parameters in all the groups of rats. **(A)** The correlation between the altered-OTUs and host metabolic parameters between the HFD (14W) and HFD + CUR (14W) groups. **(B)** The correlation between the altered-OTUs and host metabolic parameters between the HFD (14W) and HFD + MET (14W) groups. Rows correspond to OTUs with the identities shown on the right and columns correspond to metabolic parameters. Red and green colors indicate positive and negative association, respectively. The intensity of the colors represents the degree of association between the OTU abundances and host parameters as assessed by the Spearman’s correlations analysis. The asterisks indicate that the associations were significant. The taxonomy of the OTUs is shown on the left.

To further analyze the relationship between gut microbiota and metabolic parameters associated with NAFLD rats, Spearman correlation analysis was used to identify specific gut bacteria that potentially mediate in the beneficial effects of CUR and MET on hepatic steatosis and inflammation. Between HFD (14W) and HFD + CUR (14W) groups, *Helicobacter* (OTU21), *Akkermansia* (OTU20), *Corynebacterium* (OTU226), *Lactobacillus* (OTU5,OTU239), *Ruminococcaceae_unclassified* (OTU156), *Achnospiraceae_unclassified* (OTU473), *Corynebacterium* (OTU226), and *Clostridiales_unclassified* (OTU12) exhibited a significant positive correlation with TG, TC, LDL-C, IL-6, IL-1β, and TNF-α, and a negative correlation with HDL-C (*p* < 0.05). However, *Ruminococcus* (OTU252), *Lachnospiraceae_ unclassified* (OTU220, OTU616, OTU67, OTU125, OTU63), *Porphyromonadaceae_unclassified* (OTU35, OTU43, OTU1430, OTU261), *Ruminococcaceae_unclassified* (OTU113, OTU261), *Stomatobaculum* (OTU215) and *Firmicutes_unclassified* (OTU165) exhibited a significantly negative correlation with ALT, AST, TC, TG, LDL-C, GLU, IL-6, IL-1β, TNF-α, IL-23,IP-10, and OX40 (*p* < 0.05) (Figure 12A). Comparison between HFD (14W) and HFD + MET (14W) groups showed that *Desulfovibrio* (OTU17), *Romboutsia* (OTU9), *Ruminococcaceae_unclassified* (OTU156), and *Corynebacterium* (OTU226) were positively correlated with ALT, IL-6, IL-1β, TNF-α, IL-23, IP-10, and OX40. *Prevotella* (OTU2, OTU558, OTU656), *Lachnospiraceae_unclassified* (OTU67) and *Firmicutes_ unclassified* (OTU263) exhibited a significant negative correlation with ALT, IL-6, IL-1β, TNF-α, IL-23, IP-10, and OX40 (*p* < 0.05) (Figure 12B).

## Discussion

Previous studies suggested that an increase in the *Firmicutes-to-Bacteroidetes* (F/B) ratio was closely related to obesity (Mathur and Barlow, 2015; Fontané et al., 2018). It was found in our study that the gut microbiota of obese rats underwent obvious changes after HFD feeding, and the F/B ratio was significantly increased in HFD (10W) group compared with that in HFD (0W) group. Major significant findings in this experiment included an increase in the abundance of *Firmicutes, Actinobacteria* and *Proteobacteria* and a decrease in *Bacteriodetes* and *Tenericutes* at the phylum level in HFD-induced obese rats. Consistent with the study by Velazquez et al. (2019), we also observed changes in *Firmicutes, Actinobacteria, Bacteriodetes*, and *Tenericutes* in HFD-induced rats, probably due to changes in the body composition. Özkul et al. (2017) reported that *Proteobacteria* over-presence was related to endotoxemia and the pathogenesis of NAFLD. The abundance of *Blautia, Allobaculum, Desulfovibrio, Anaerostipes, Roseburia*, and *Romboutsia* was enriched markedly at the genus level in HFD (10W) group as compared with HFD (0W) group. Several studies have shown that the genera of *Blautia* and *Roseburia* are correlated with obesity development, which may explain an unfavorable impact on health. For example, Lin et al. (2019) discovered that an increased abundance in genera *Blautia* and *Roseburia* was often accompanied with an increase in deoxycholic acid and taurodeoxycholic acid levels, which may represent gut microbiota-host cometabolism in the HFD-induced obese rat model. Zeng et al. (2019) performed a clinical study and found that *Blautia*, *Romboutsia*, *Ruminococcus*, and *Dorea* were positively correlated with indicators of bodyweight and serum lipids. In addition, Hu et al. (2019) showed that both *Romboutsia* and *Desulfovibrio* were obesity-related bacteria. Li et al. (2018) reported a significant relationship between *Romboutsia* and serum TC, TG, and LDL-C levels. Our experiment also showed a positive correlation of *Desulfovibrio* and *Romboutsia* with ALT, TG, and some inflammatory factors including IL-6, IL-1β, TNF-α, IL-23, IP-10, and OX40. Some recent studies (Cox et al., 2014; Chen et al., 2018; Van Hul et al., 2018) suggested that *Allobaculum*, which belongs to the SCFA-producing bacteria, could prevent the onset of obesity in mice. But we found unexpectedly that the abundance of *Allobaculum* was increased in HFD (10W) group, which may be relative to the use of adult rats in this study. In addition, we also found that the genera *Anaeroplasma* and *Alistipes* in HFD (10W) group were less abundant compared with those in HFD (0W) group. Granado-Serrano et al. (2019) reported a lower relative abundance of *Anaeroplasma* in males with hypercholesterolemia. *Alistipes*, which belong to the family *Rikenellaceae*, were also found to be reduced in NAFLD patients (Zhu et al., 2013).

To clarify the role of gut microbiota change in the anti-NAFLD process mediated by CUR and MET, we made a direct comparison between them and found that the F/B ratio was decreased after CUR and MET intervention. In addition, PCoA showed that both CUR and MET reverted the gut microbiota structure altered by HFD, which is consistent with the finding of previous related studies (Zhang et al., 2015; Feng et al., 2017). Analysis of bacterial OTUs showed that a total of 129 OTUs showed similar response to the intervention of the two drugs. Among the 129 OTUs, 45 and 57 abundant ones were significantly altered after CUR and MET treatment, respectively. Both CUR and MET increased the abundance of *Lactobacillus, Lactococcus, Bacteroides, Clostridium_XIVb, Ruminoccoccaceae_unclassified, Porphyromonadaceae_ unclassified*, and *Lachnospiraceae_unclassified. Bacteroides, Lactobacillus, Clostridium XIVb*, and *Ruminoccoccaceae* are all beneficial bacteria which are producers of SCFAs as fermentation end-products of proteins and carbohydrates (Cummings and Macfarlane, 1991). Other studies also found similar alterations in gut microbiota (Zhang et al., 2015, 2019; Feng et al., 2017), suggesting that the SCFA-producing bacteria play a crucial role in the pharmacology of both CUR and MET. Previous studies have already indicated an essential role of SCFAs in the gastrointestinal system, particularly in actively modulating host fat mass storage and treating obesity, DM2 and even colorectal cancer (D’Argenio et al., 1996; Galvez et al., 2005; Murphy et al., 2010; Qin et al., 2012; Smith et al., 2013; Xu et al., 2015). In addition, SCFA-producing bacteria also exhibited a positive impact on the host through protecting the mucosa against pathogens, providing colonocytes with nutrients, and reducing inflammation (Maslowski et al., 2009; Neyrinck et al., 2011). Tang et al. (2018) reported that a traditional Chinese herbal formula (HuganQingzhi Tablets, HQT) could effectively recover the abundance of SCFA-producing bacteria such as *Ruminococcaceae* during the treatment of rats with NAFLD induced by HFD. Furthermore, previous studies on CUR also showed that the SCFA-producing bacteria including *Allobaculum, Bacteroides, Phascolarctobacterium*, and *Blautia* in the rat gut were enriched markedly during CUR treatment of HFD-induced obesity rats (Feng et al., 2017). Therefore, the selective regulation of specific types of the gut microbes, especially the enrichment of SCFA producing bacteria, may be involved in the role of CUR and MET in alleviating host metabolic diseases.

In addition to the increase of SCFA-producing bacteria, CUR and MET showed high similarity in inhibiting a variety of intestinal microbes. Previous studies reported that the antimicrobial activity of CUR in the gut was related to its anti-obesity effect (Jin et al., 2018). For example, a study (Saberi-Karimian et al., 2020) reported that CUR (200 mg/kg) could alleviate liver fibrosis in male rats by inhibiting the expression of inflammatory cytokines such as TNF-α, IL-6, and MCP-1 and eventually prevent the development and progression of obesity-related diseases. Sun et al. (2018) also demonstrated that MET exerted its hyperglycemia-lowering effect by altering the community of gut microbiota. All these previous investigations suggest that both CUR and MET could suppress the growth of pathogens related to diet-induced metabolic disorders. In this experiment, we identified that the abundance of *Acinetobacter, Sphingomonas, Porphyromonadaceae_unclassified, Coprobacillus, Ruminococcus*, and *Staphylococcus* was decreased after CUR or MET treatment, and some of these genera were increased by HFD. In addition, we observed the drug-specific regulations of gut microbiota by CUR and MET. Consistent with previous studies (Shin et al., 2014), some beneficial bacteria such as *Prevotella* were increased in rats treated with MET but not in rats treated with CUR.

It was found in our study that *Butyricicoccus*, a butyrate-producing genus in the intestinal tract, was markedly enriched after CUR treatment. Some other recent studies (Rodriguez et al., 2020) reported that *Butyricicoccus* could decrease the body mass index (BMI) in obese individuals. We also found that CUR decreased the relative abundance of *Dorea* in rats, which is known to be related to the development of obesity both in rats and children (Karvonen et al., 2019; Velazquez et al., 2019). Collectively, CUR was able to prevent HFD-induced NAFLD in multiple ways, indicating that CUR may have gut microbiota as its main drug target during the treatment of NAFLD. Nevertheless, further evidence is needed to determine the mechanism of CUR in modulating gut microbiota in the context of NAFLD.

## Conclusion

In conclusion, this study demonstrated that both CUR and MET could reduce liver steatosis and modulate gut microbiota. Both drugs were able to attenuate liver inflammation and improve the intestinal barrier. Observations of the gut microbiota showed that CUR and MET positively affected imbalances in obese rats by upregulating the abundance of beneficial bacteria and downregulating the abundance of opportunistic pathogens.

An overall review of the above results suggests that CUR and MET are effective in modulating liver inflammation, improving intestinal integrity, controlling weight gain and serum lipid metabolism, and supporting a healthy structure of gut microbiota. These findings suggest that CUR and MET may prove to be effective treatments for NAFLD patients. However, there are still shortcomings in this study. For example, the feces of normal rats have not been completely collected so that they can’t be used to benchmark the efficacy of CUR and MET. The mechanism by which these drugs specifically modulate the gut microbiota composition remains unclear and more studies are required to further explore the mechanism of the CUR/MET-gut microbiota-intestinal epithelium axis as the therapeutic target of NAFLD.

## Data Availability Statement

The raw Illumina read data for all samples were deposited in the European Bioinformatics Institute European Nucleotide Archive database under the accession number PRJNA678147.

## Ethics Statement

The animal study was reviewed and approved by the Research Animal Care Committee of Jinshan Hospital affiliated to Fudan University, China.

## Author Contributions

SB and PG designed and guided the study. RL and YY performed the induction of NAFLD rat model and drug administration. RL performed the experiment and wrote the manuscript. All authors commented on and approved the manuscript.

## Conflict of Interest

The authors declare that the research was conducted in the absence of any commercial or financial relationships that could be construed as a potential conflict of interest.
