# Supplementary material for: The Therapeutic Efficacy of Curcumin vs. Metformin in Modulating the Gut Microbiota in NAFLD Rats: A Comparative Study
Source: Front Microbiol. 2021 Jan 14;11:555293. doi: 10.3389/fmicb.2020.555293 (PMC7874275; doi:10.3389/fmicb.2020.555293)

Supplementary Materials

Fgiure S1:Histological analysis of HFD-induced hepatic steatosis.

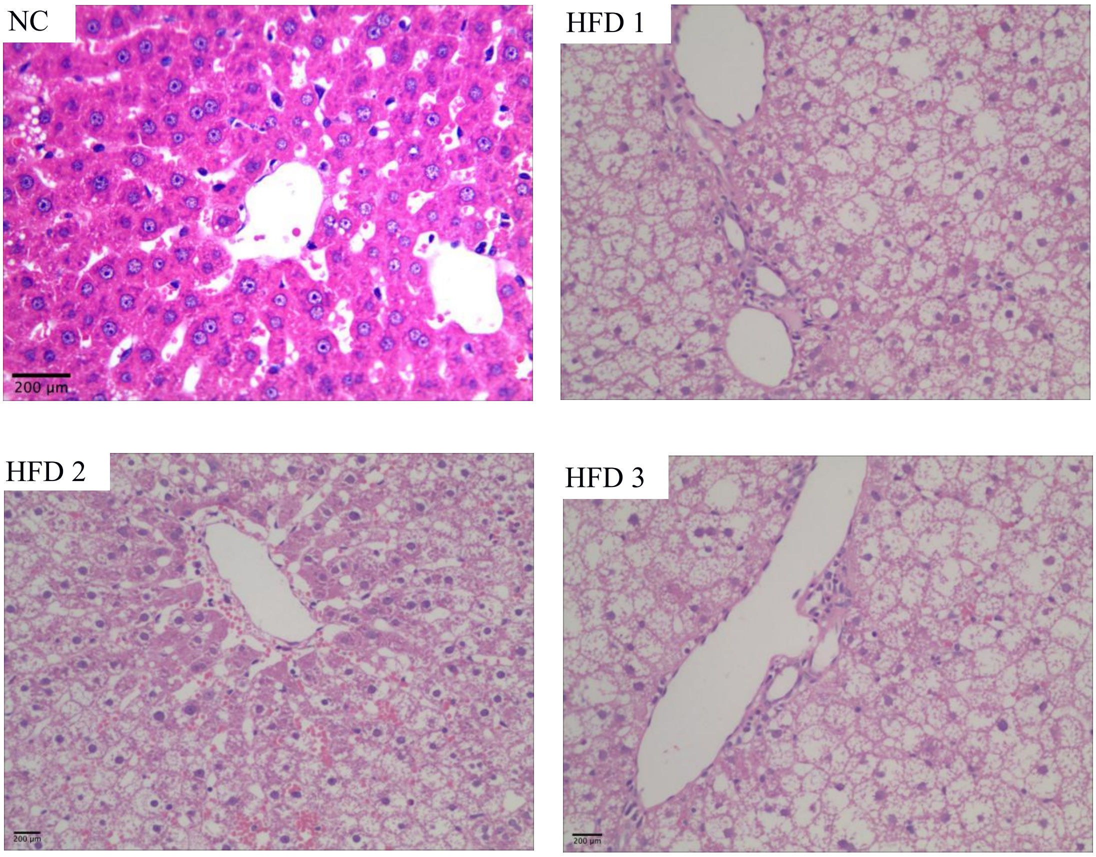

the HE-stained liver tissue(400X)

Table S1: NAFLD activity score of the liver tissue under HFD -induced obesity

Parameters	NC	HFD 1	HFD 2	HFD 3
Steatosis (0-2)	0	2	1	2
Lobular inflammation (0-3)	0	1	2	1
Ballooning degeneration (0-2)	0	2	1	2
Total NAS(0-8)	0	5	4	5

Table S2: The community coverage index of each fece sample.

Feeding Method	Time	Sample ID	0.97 (The operational taxonomic units(OTUs) that reached a 97% nucleotide similarity level)					
			OTU	ace	chao	coverage	shannon	simpson
HFD	0W	C011	613	705 (677,747)	708 (672,765)	0.996432	4.57 (4.55,4.59)	0.0337 (0.0327,0.0347)
		C012	536	647 (614,695)	631 (596,687)	0.995405	4.4 (4.38,4.42)	0.0276 (0.027,0.0282)
		C014	659	748 (720,787)	776 (733,845)	0.996755	4.56 (4.54,4.58)	0.0302 (0.0295,0.031)
		C015	666	766 (736,808)	783 (741,848)	0.99577	4.71 (4.69,4.73)	0.0225 (0.0219,0.023)
		C06	685	798 (766,844)	786 (750,841)	0.996595	4.51 (4.49,4.52)	0.03 (0.0293,0.0306)
		C07	605	714 (681,760)	736 (688,812)	0.995905	4.33 (4.3,4.35)	0.052 (0.0505,0.0536)
		H013	800	895 (866,935)	897 (862,951)	0.99832	4.7 (4.69,4.71)	0.0228 (0.0224,0.0231)
		H016	630	718 (690,757)	711 (680,760)	0.99876	3.91 (3.9,3.93)	0.0507 (0.0501,0.0513)
		H019	696	823 (788,871)	829 (784,898)	0.997879	3.56 (3.55,3.58)	0.0847 (0.0835,0.0859)
		H024	738	879 (840,932)	897 (843,979)	0.997984	4.2 (4.19,4.21)	0.0464 (0.0457,0.0471)
		H08	640	729 (702,769)	713 (686,757)	0.997989	4.04 (4.03,4.06)	0.0545 (0.0536,0.0555)
		H09	799	870 (847,903)	867 (840,911)	0.999264	4.32 (4.31,4.33)	0.0356 (0.0352,0.036)
		M017	645	762 (728,809)	784 (736,858)	0.995393	4.33 (4.31,4.35)	0.0439 (0.0427,0.0452)
		M02	670	811 (772,864)	825 (773,904)	0.994945	4.61 (4.59,4.63)	0.0256 (0.025,0.0263)
		M020	610	773 (729,833)	751 (705,819)	0.994687	4.01 (3.99,4.04)	0.0529 (0.0517,0.0542)
		M022	598	723 (687,774)	735 (687,809)	0.994609	4.48 (4.46,4.5)	0.0272 (0.0265,0.0278)
		M023	564	678 (645,725)	678 (638,741)	0.992933	4.38 (4.35,4.4)	0.0353 (0.034,0.0366)
		M05	663	753 (726,792)	777 (736,843)	0.996721	4.32 (4.3,4.33)	0.0457 (0.0445,0.0469)

HFD	10W	H713	596	698 (667,742)	693 (658,748)	0.998062	3.67 (3.65,3.69)	0.0777 (0.0766,0.0789)
		H716	587	687 (656,732)	682 (646,738)	0.998541	3.96 (3.94,3.97)	0.0524 (0.0517,0.0532)
		H719	519	631 (597,679)	628 (588,691)	0.997873	3.45 (3.44,3.47)	0.0877 (0.0863,0.089)
		H724	554	614 (593,646)	603 (582,639)	0.999112	3.73 (3.72,3.74)	0.0941 (0.0927,0.0956)
		H78	734	776 (760,801)	776 (757,810)	0.999434	4.65 (4.64,4.66)	0.0208 (0.0205,0.021)
		H79	755	856 (826,899)	857 (820,914)	0.998728	4.66 (4.66,4.67)	0.0232 (0.0229,0.0236)
		M717	533	649 (614,700)	664 (616,741)	0.996636	3.98 (3.95,4)	0.0763 (0.0742,0.0784)
		M72	457	547 (517,590)	556 (517,621)	0.997361	3.33 (3.31,3.35)	0.1335 (0.1308,0.1362)
		M720	477	597 (559,652)	650 (583,759)	0.996316	3.72 (3.69,3.74)	0.0921 (0.09,0.0943)
		M722	536	641 (608,688)	671 (620,753)	0.996929	4.05 (4.03,4.07)	0.0601 (0.0585,0.0617)
		M723	609	762 (719,821)	775 (719,859)	0.994961	3.95 (3.93,3.98)	0.0997 (0.0968,0.1026)
		M75	404	500 (468,549)	515 (470,591)	0.997715	3.31 (3.29,3.33)	0.1255 (0.1227,0.1282)
		C711	548	676 (639,730)	685 (636,762)	0.996546	3.4 (3.37,3.42)	0.1609 (0.1573,0.1644)
		C712	453	557 (523,606)	556 (516,623)	0.998207	3.91 (3.9,3.93)	0.0414 (0.0408,0.0419)
		C714	535	667 (627,724)	704 (641,804)	0.998352	3.9 (3.89,3.92)	0.0615 (0.0604,0.0627)
		C715	392	499 (463,552)	497 (455,567)	0.996645	3.76 (3.75,3.78)	0.053 (0.0519,0.0541)
		C76	442	555 (519,608)	571 (520,655)	0.997692	3.55 (3.53,3.58)	0.0795 (0.0781,0.0808)
		C77	527	659 (620,715)	631 (594,690)	0.996501	3.92 (3.9,3.94)	0.0742 (0.0723,0.0762)
HFD	14W	H121	676	747 (724,781)	744 (717,788)	0.998713	4.39 (4.38,4.4)	0.0379 (0.0372,0.0387)
		H121	698	785 (758,825)	802 (762,865)	0.998692	4.81 (4.8,4.82)	0.0148 (0.0146,0.0149)
		H121	647	763 (729,810)	741 (707,793)	0.997812	4.14 (4.12,4.15)	0.0471 (0.0463,0.048)

		H122	516	600 (573,641)	571 (549,608)	0.998396	3.84 (3.82,3.85)	0.0727 (0.0713,0.0742)
		H128	641	708 (686,742)	704 (678,747)	0.999044	4.57 (4.56,4.58)	0.0217 (0.0214,0.0219)
		H129	567	679 (646,726)	677 (637,739)	0.994987	4.41 (4.39,4.43)	0.0257 (0.0252,0.0263)
HFD+MET	14W	M1217	566	667 (636,712)	695 (647,772)	0.996125	4.1 (4.08,4.12)	0.066 (0.0641,0.068)
		M122	524	622 (592,665)	615 (581,670)	0.996002	3.65 (3.62,3.68)	0.1324 (0.1287,0.136)
		M1220	585	679 (650,722)	685 (647,744)	0.995785	4.68 (4.66,4.71)	0.0297 (0.0286,0.0308)
		M1222	536	654 (619,704)	663 (617,735)	0.994444	4.08 (4.06,4.11)	0.0578 (0.0561,0.0594)
		M1223	637	742 (710,786)	751 (710,817)	0.996033	4.48 (4.46,4.5)	0.0355 (0.0345,0.0365)
		M125	577	693 (659,742)	706 (659,779)	0.995431	4.22 (4.19,4.24)	0.0599 (0.0579,0.0619)
HFD+CUR	14W	C121	595	712 (677,762)	742 (688,825)	0.996522	4.14 (4.12,4.16)	0.0693 (0.0674,0.0713)
		C121	609	694 (667,734)	729 (682,806)	0.997252	4.85 (4.84,4.86)	0.0156 (0.0153,0.0159)
		C121	615	716 (685,760)	730 (688,798)	0.995364	4.91 (4.89,4.93)	0.0142 (0.0139,0.0145)
		C121	609	683 (659,719)	687 (656,737)	0.997038	5.06 (5.05,5.07)	0.0109 (0.0107,0.0111)
		C126	651	743 (715,783)	733 (702,783)	0.996218	4.77 (4.75,4.78)	0.0237 (0.0229,0.0245)
		C127	647	771 (736,820)	784 (736,858)	0.994986	4.52 (4.5,4.54)	0.044 (0.0424,0.0455)

The two numbers surrounded by parentheses are minimal and maximal degrees output by multiple analyses.

Figure S2: The Alpha diversity of each group. (A) The observer OTUs of each group. (B) The ace index of each group. (C) The chao index of each group. (D) The shannon index of each group. (E)The simpson index of each group.

A

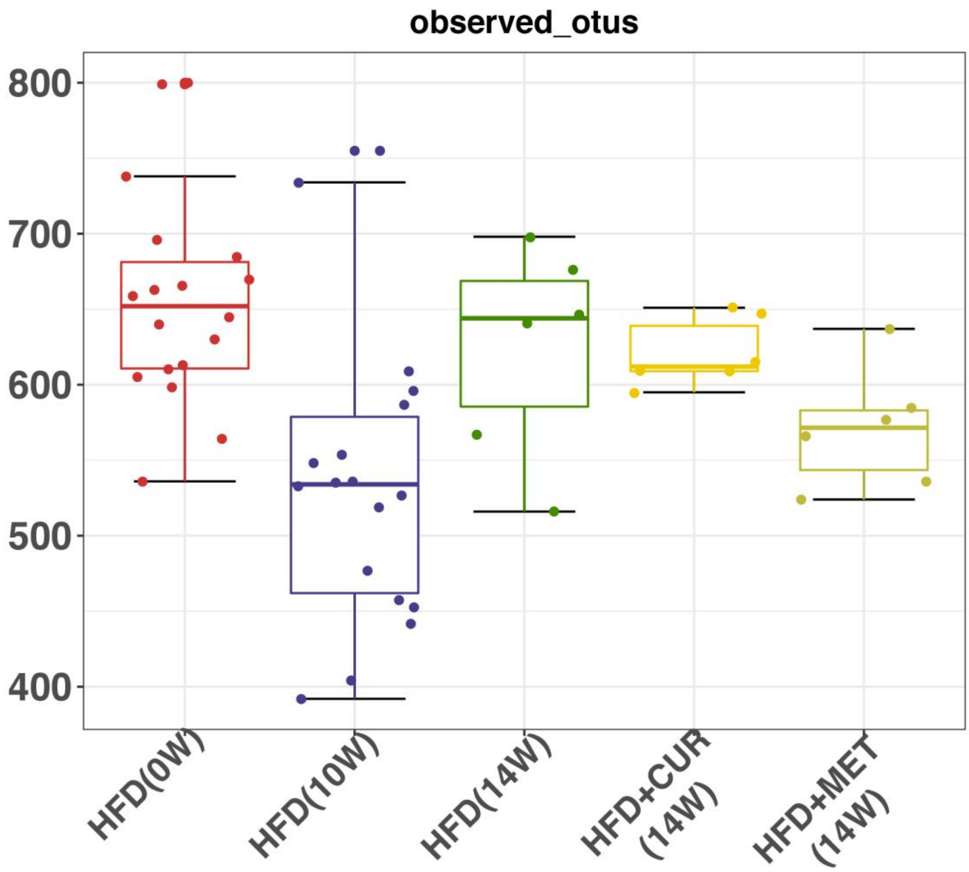

B

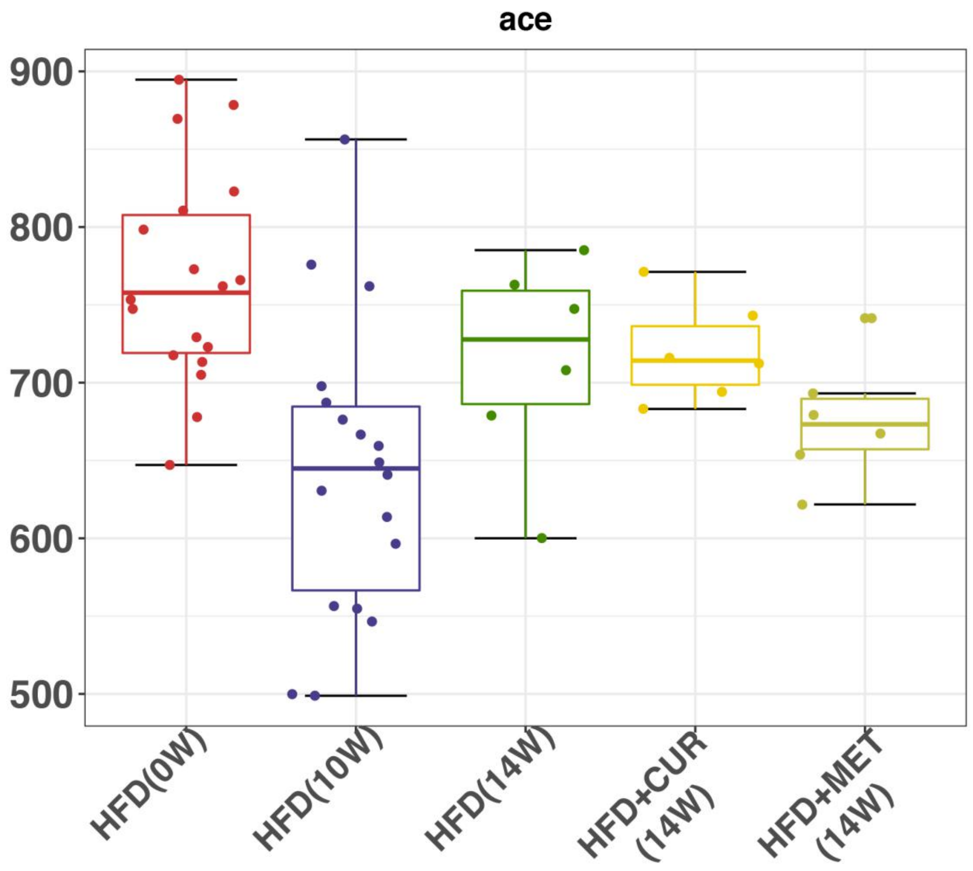
ace
900
800
700
600
500
HFD(0W)
HFD(10W)
HFD(14W)
HFD+CUR (14W)
HFD+MET (14W)

C

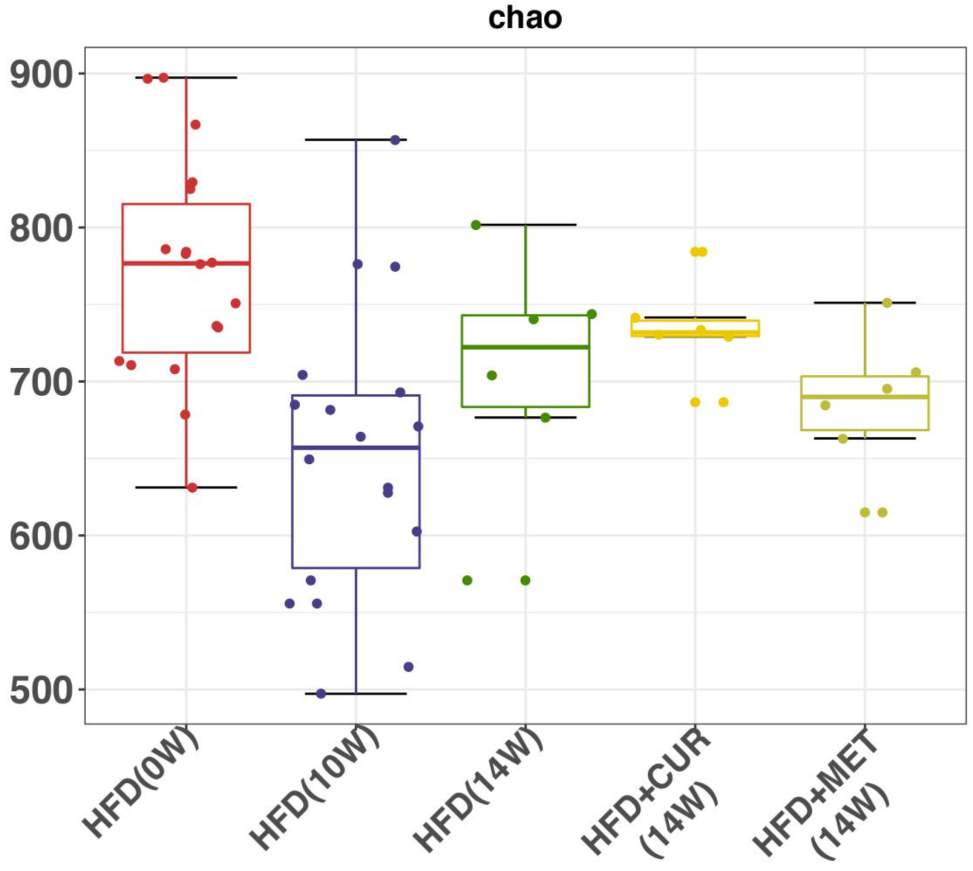
chao
900
800
700
600
500
HFD(0W)
HFD(10W)
HFD(14W)
HFD+CUR (14W)
HFD+MET (14W)

D

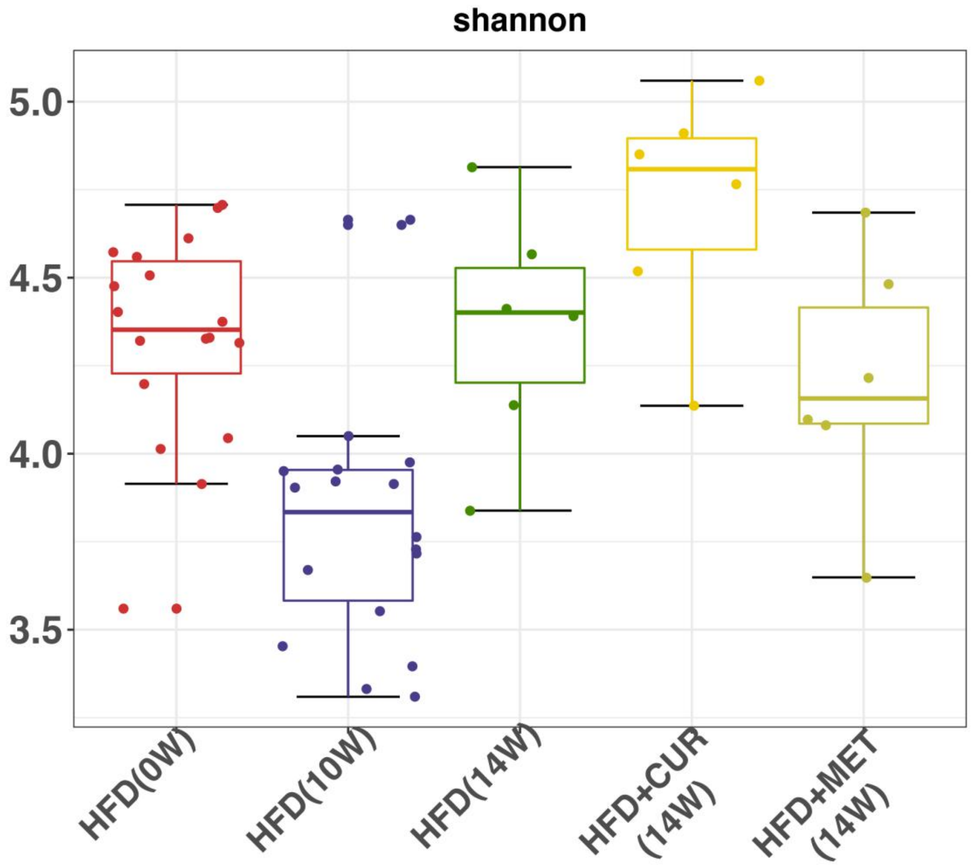
shannon
5.0
4.5
4.0
3.5
HFD(0W)
HFD(10W)
HFD(14W)
HFD+CUR (14W)
HFD+MET (14W)

E

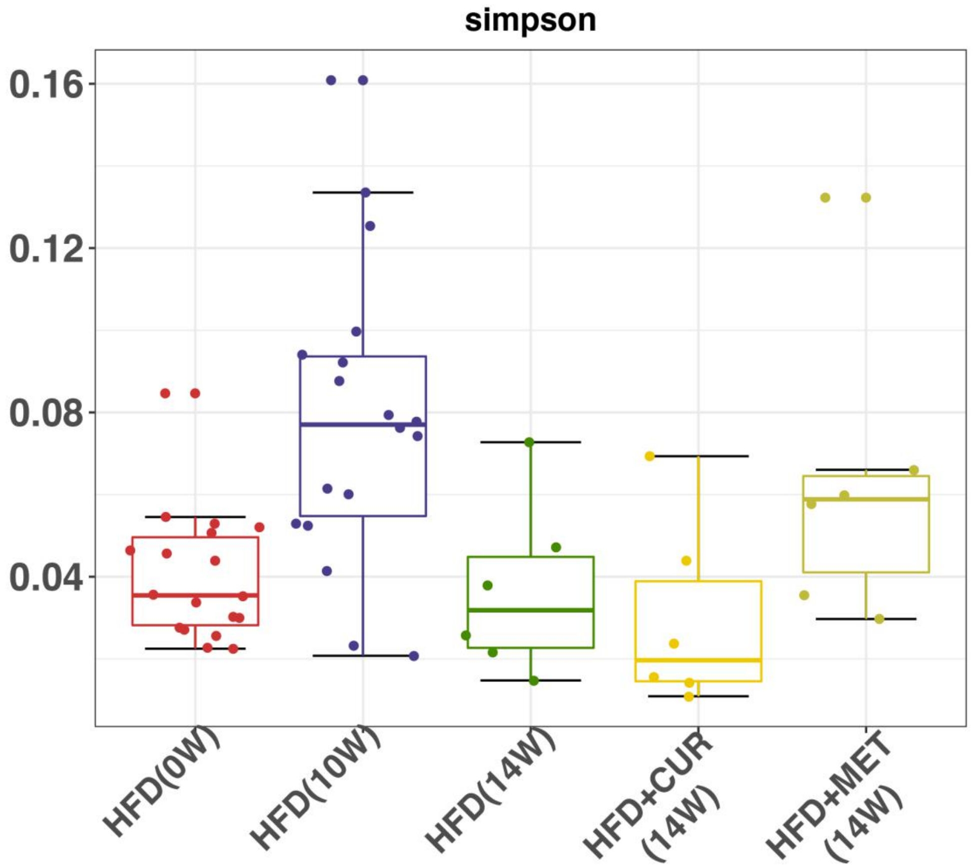
simpson
0.16
0.12
0.08
0.04
HFD(0W)
HFD(10W)
HFD(14W)
HFD+CUR (14W)
HFD+MET (14W)

Figure S3: The rarefaction curves and Shannon curves of each fece sample. (A) The rarefaction curves of each fece sample. (B) The Shannon curves of each fece sample.

A

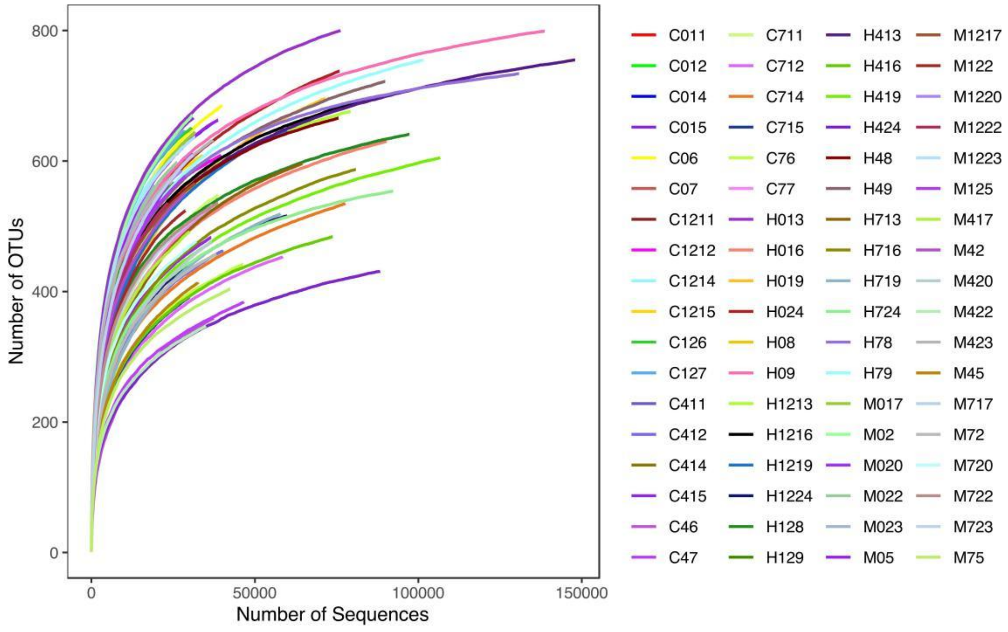

B

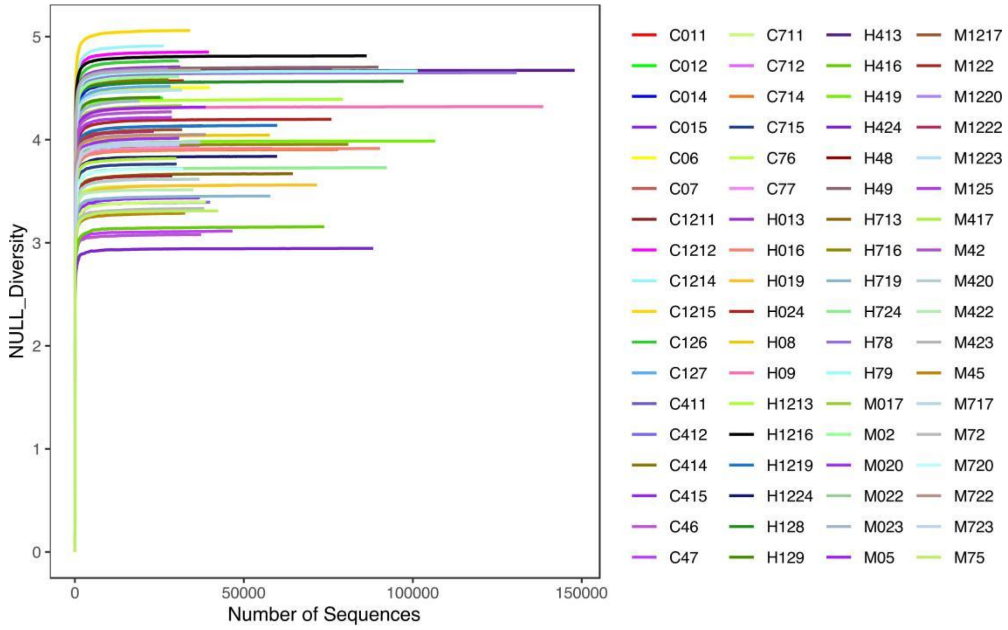

Figure S4: PCoA score plot based on weighted Unifrac metrics.

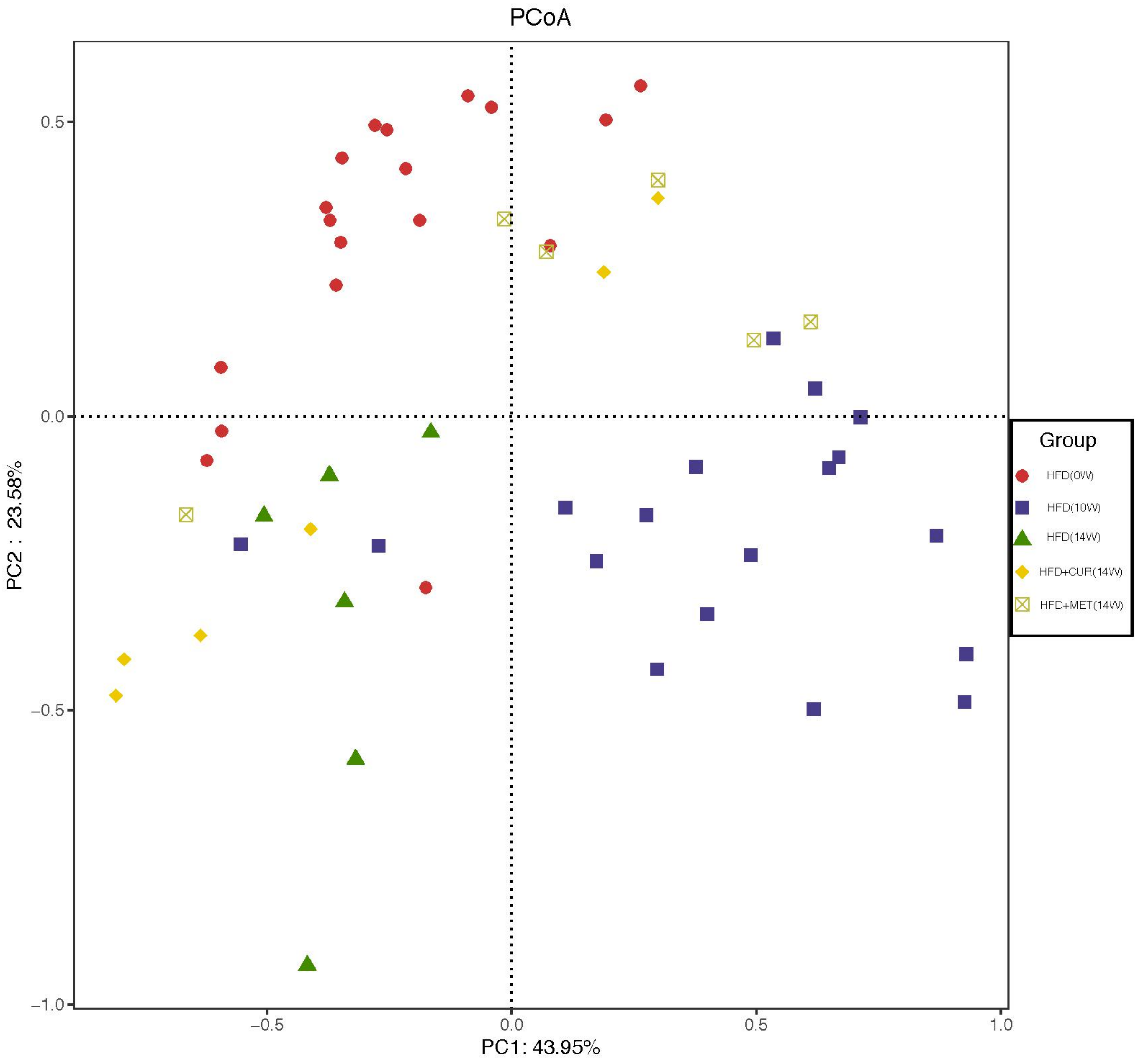

Figure S5: Heatmap of the abundance the OTUs in the HFD(0W), HFD (10W), HFD (14W), HFD+CUR(14W) and HFD+MET(14W) groups.

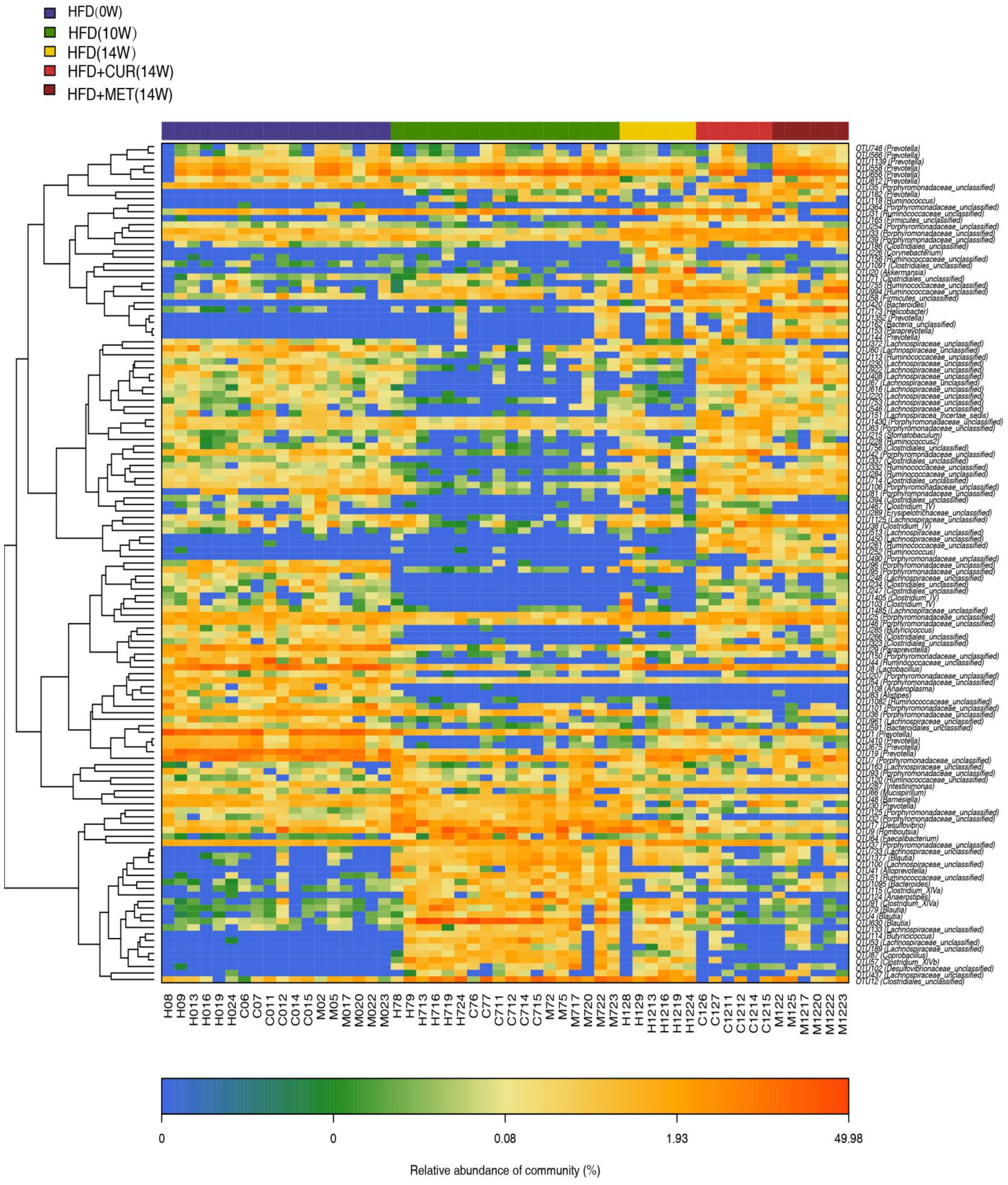

Supplement: Supplementary file 1 [file Data_Sheet_1.pdf]
